# Understanding the Effect of Dispersant Rheology and Binder Decomposition on 3D Printing of a Solid Oxide Fuel Cell

**DOI:** 10.3390/mi15050636

**Published:** 2024-05-09

**Authors:** Man Yang, Santosh Kumar Parupelli, Zhigang Xu, Salil Desai

**Affiliations:** 1 Industrial and Systems Engineering, North Carolina A & T State University, Greensboro, NC 27411, USA; amandayang2005@gmail.com (M.Y.); sparupel@ncat.edu (S.K.P.); 2 Center of Excellence in Product Design and Advanced Manufacturing, North Carolina A & T State University, Greensboro, NC 27411, USA; 3 Mechanical Engineering, North Carolina A & T State University, Greensboro, NC 27411, USA; zhigang@ncat.edu

**Keywords:** 3D printing, binder concentration, dispersant, rheology behavior, solid-oxide fuel cells

## Abstract

Solid oxide fuel cells (SOFCs) are a green energy technology that offers a cleaner and more efficient alternative to fossil fuels. The efficiency and utility of SOFCs can be enhanced by fabricating miniaturized component structures within the fuel cell footprint. In this research work, the parallel-connected inter-digitized design of micro-single-chamber SOFCs (µ-SC-SOFCs) was fabricated by a direct-write microfabrication technique. To understand and optimize the direct-write process, the cathode electrode slurry was investigated. Initially, the effects of dispersant Triton X-100 on LSCF (La0.6Sr0.2Fe0.8Co0.2O3-δ) slurry rheology was investigated. The effect of binder decomposition on the cathode electrode lines was evaluated, and further, the optimum sintering profile was determined. Results illustrate that the optimum concentration of Triton X-100 for different slurries was around 0.2–0.4% of the LSCF solid loading. A total of 60% of solid loading slurries had high viscosities and attained stability after 300 s. In addition, 40–50% solid loading slurries had relatively lower viscosity and attainted stability after 200 s. Solid loading and binder affected not only the slurry’s viscosity but also its rheology behavior. Based on the findings of this research, a slurry with 50% solid loading, 12% binder, and 0.2% dispersant was determined to be the optimal value for the fabricating of SOFCs using the direct-write method. This research work establishes guidelines for fabricating the micro-single-chamber solid oxide fuel cells by optimizing the direct-write slurry deposition process with high accuracy.

## 1. Introduction

The dramatic convergence of global economic evolution, technological advancements, a fast-growing population, and social improvements have escalated the demand for energy resources [1,2,3]. Currently, there exist several green methods [4,5] to produce power which include solar power [6], hydropower [7], wind power [8], nuclear power [9], geothermal power [10], biodiesel [11], etc. Nevertheless, the above-mentioned power generation techniques have distinct limitations and drawbacks. Therefore, there is a significant need for the development of novel ways of generating power. Fuel cells, an advanced prospective green energy solution, is obtaining eminence importance for producing power [12,13]. A fuel cell consists of an unglazed earthenware electrolyte and gold/platinum foil electrodes [14,15]. Fuel cells, a green energy technology, have the inherent ability to utilize minimal fuel more energy efficiently than the above-mentioned competing technologies with minimal or negligible pollutant discharges into the environment [16,17,18,19,20]. Generally, a fuel cell consists of three components which include two electrodes—an anode (positive) and a cathode (negative), as well as an electrolyte.

Currently, the highest-temperature fuel cells in development are solid oxide fuel cells which can be operated over a wider temperature range from 600 °C to 1000 °C, allowing for several fuels to be used. Solid oxide, or solid ceramic material, is the electrolyte for high-temperature activities because it conducts oxygen ions (O_2_). Along with an operational efficiency of over 60%, this fuel cell has one of the highest rates of energy generation compared to other energy conversion devices [21,22]. This is enabled due to the high operational temperature of SOFCs enabling cogeneration systems to create high-pressure steam, which can be utilized in multiple applications. The development of portable electronics and micro-electromechanical systems (MEMSs) has made miniature fuel cells desirable options for power sources. Micro-solid oxide fuel cells provide shorter recharge times and greater energy densities than traditional electrochemical batteries [23,24,25,26,27,28].

Researchers are investigating various microfabrication procedures to fabricate µ-SOFCs [29,30,31] with enhanced efficiency [32,33,34,35]. To reduce the ohmic losses in the electrolyte, micropatterning technologies can be used, where electrodes are placed closer together. Moreover, by applying microfabrication, the single-chamber solid oxide fuel cell (SC-SOFC) architecture can be coupled in series or parallel arrays to enhance the overall voltage, current, and power of the assembly. Hibino (1996), the pioneer of SC-SOFC, created a new cell design with an interpenetrating comb-like electrode pattern on the same surface of an electrolytic material with feature sizes in the millimeter range [36]. Chung and Chung applied finite element modeling to simulate a micro-single-chamber solid oxide fuel cells (µ-SC-SOFCs) with a side-by-side architecture [37]. The research aimed to enhance the efficiency and lower the operating temperature of µ-SC-SOFCs. Most studies have used a co-planar configuration to investigate the properties, operating parameters, and output power of various electrodes and electrolyte materials [38,39]. In the direct writing process [40,41,42], the suspension filament is extruded onto the substrate, which is appropriate for mass production. The suspension can be solidly loaded up to 60% using this approach. The suspension has a higher viscosity than the soft lithography solution. The initiation of the direct ink writing technique for the fabrication of three-dimensional ceramic pieces without depending on costly tooling dies or lithographic masks was spearheaded by Prof. Jennifer A. Lewis [43,44]. Employing this technology enables the fabrication of intricate three-dimensional shapes with custom design patterns without expensive and skilled tooling and masking. For applications in the structural, functional, and biological domains, three-dimensional (3D) ceramic material patterning is crucial [45,46]. Yong Bum Kim [47] effectively used the direct write method in 2005 to build integrated planar solid oxide fuel that was serially coupled. The anode (YSZ + NiO) and cathode (LSCF) were directly written on the side of a zirconia substrate that was moderately stabilized with Yttria. Sung-Jin Ahn et al. [48] extruded the cathode (LSM) and anode (NiO-SDC-Pd) using a syringe nozzle onto the YSZ substrate in the year 2006 using the direct write technique. Anode and cathode widths were approximately 500 µm and 450 µm, individually. The direct write technique was utilized to fabricate parallel-connected SC-SOFC on an electrolyte pie, involving a method where the electrolyte width exceeded 500 µm, as reported by Melanie Kuhn’s research group [49,50,51,52] in 2007. With a mean electrode gap of about 1 mm, the experiment determined that both cells maintained stable open circuit voltage at 0.9 V and attained a peak power density of 2.3 mWcm^−2^.

There exist several slurry compositions with a good level of rheological analysis; however, our research focuses on understanding the composition and rheology of a specific slurry composition for LCSF-based cathode electrodes. Ali et al. [53] research study focused on evaluating LSCF ink composition for powder and electrochemical characterization in screen printing applications. It is important to note that our work focuses on 3D printing of an LSCF cathode electrode which has different rheological requirements compared to screen printing applications. Moreover, the authors in the above-mentioned publication did not evaluate any rheological behavior of slurry which is critical in our case given the 3D printing application. Finally, 3D printing permits high solid loading of 40 to 60% and thus controlled porosity of microstructures as compared to very low viscosity and solid loading content in screen printing inks. Thus, though there are several slurry products and similar compositions in the literature, they do not address important rheological properties required in 3D printing of an LSCF cathode electrode, which our research comprehensively addresses.

It is very crucial to understand the effect of the dispersant, slurry loading, and binder on the direct-write process as it has a significant impact on the fabricated SOFCs. In our research, the focus is on fabricating parallel-connected inter-digitized design µ-SC-SOFCs. The cathode electrode slurry was investigated to understand and optimize the direct-write technique. Initially, the effects of dispersant on LSCF (La0.6Sr0.2Fe0.8Co0.2O3-δ) slurry rheology were investigated. Further, the binder removal process was investigated, and the optimum sintering profile was determined as it has a substantial impact on fuel cell microstructure.

## 2. Materials and Methods

**Figure 1:**
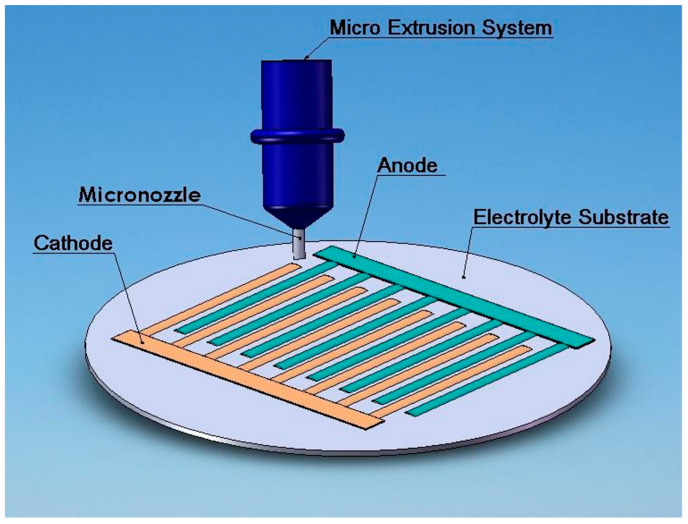
Schematic illustration of a SC-SOFC in coplanar design and process.

The direct-writing process of the SC-SOFC in coplanar design is illustrated in Figure 1 as reported in the previous research work [54]. The direct writing system consists of a micro extrusion system, a sample holder, a pressure regulator, and a motion controller. The micro extrusion system incorporates an air cylinder, a reservoir retainer, piston, and a micronozzle. This technique consists of various sequential steps. Initially, the electrolyte substrate is prepared; then, the anode slurry is loaded into the reservoir retainer, which is held in an air cylinder. The air pressure for the piston loaded on the end of the retainer is regulated. By altering the pressure, the slurry is extruded from the micronozzle. Concurrently, the velocity, acceleration, and deceleration of the motion stage are regulated by the motion controller. Consecutive lines of the anode are created on the electrolyte substrate. After that, the platinum wire is attached to the anode slurry, and the annealing of anode lines is completed. Similarly, the cathode slurry is loaded in the reservoir and handled in the above-mentioned manner to build the cathode lines. Finally, the fuel cell is examined to determine its performance [54].

The custom microstructure of the anode and cathode can be fabricated by controlling the air pressure, velocity, the mixture ratio of the slurry, and the distance between the nozzle and the substrate. After annealing, the structures of printed electrodes are maintained.

### 2.1. Requirements of the Slurry for Direct Writing

Nano- or submicron-sized powders exhibit excellent potential for SOFC applications because of their low sintering temperature and high surface area with the capability of microstructure tuning when the attractive forces are substantial [55]. This study’s inclination for nano- or submicron powder sizes is corroborated by findings showcasing micro-size fuel cell production employing comparable powder sizes [56]. To accomplish relatively isotropic properties, the powder’s particle size should be notably smaller, specifically about an order of magnitude lesser than the micro component’s smallest internal dimension [57].

#### Need for Dispersant

The simultaneous presence of fine particles and high solid loading causes strong particle–particle interactions, which in turn hinders slurry management in the direct writing process. These outcomes enhance slurry viscosity. Particle size, concentration, and viscosity, the entirety of which are impacted by the addition of binder, have a greater impact on the stability and sedimentation of the slurry. Ceramic powders clump due to attractive Van der Waals forces; nevertheless, this can be prevented by employing appropriate dispersants that alter the powder’s surface properties. This permits repulsive forces (derived from steric hindrance from large-molecule adsorption or electrostatic repulsion through electrical double-layer overlap) to overcome attractive forces and maintain the dispersion of the particles [56]. A high solid content slurry’s viscosity can be considerably reduced by adding dispersants. To stabilize oxide powder slurries, these dispersants are essential in the ceramics sector. Stable, high-solid-content slurries that produce faultless, superior goods demand careful consideration of the appropriate dispersant and the right quantity for a certain ceramic powder. Lacking a dispersant, a lower-energy liquid typically instantly wets and submerges a high-energy ceramic powder surface. These changes have an abrupt effect and cannot be reversed. In contrast, the processes of dispersion and stability are more complex and take different amounts of time to unfold depending on particle density in the suspension.

### 2.2. Experiments

#### 2.2.1. Cathode Slurry Materials

The cathode material of choice was LSCF powder, which has a surface area of 6.3 m^2^/g and was supplied by the Fuel cell material company. The LSCF powders were mixed with organic solvent terpineol, dispersant: Triton X-100 (Sigma-Aldrich, St. Louis, MO, USA), and binder: Polyvinyl butyral (Sigma-Aldrich, St. Louis, MO, USA) for the formulation of the cathode slurry. Between 40 wt%, 50 wt%, and 60 wt%, the solid proportions were altered. The choice of Triton X-100 as the dispersant for stabilizing Yttrium-stabilized zirconia (YSZ) submicron powder was supported by its effective use, as registered in the literature [58]. Triton X-100 (C_14_H_22_O(C_2_H_4_O)_10_) connects a hydrophilic polyethylene oxide chain with a lipophilic or hydrophobic hydrocarbon group. It is a nonionic surfactant that normally contains 9.5 units of ethylene oxide. This compound is employed in the distribution of carbon atoms in pliable composite materials.

#### 2.2.2. Binder Concentration

It is crucial that the fuel cell electrodes incessantly retain their porous structure throughout the sintering procedure. Polyvinyl butyral (PVB) was employed as the binder for this purpose. Since PVB has a larger molecular size, combining it as a binder improves the slurry’s viscosity and stability. This advancement in viscosity helps the direct writing method achieve its envisioned purposes by progressing precision. Reaching temperatures of up to 400 °C can eliminate PVB fully [59]. In this dispersant exploration, the binder concentration was held constant at 12 wt% of the solid powder to examine the effects of the dispersant on the rheological properties of the slurry.

#### 2.2.3. Variations in Dispersant and Solid Loading Concentrations

Three distinct slurries with solid contents of 40 wt%, 50 wt%, and 60 wt% were employed. Dispersant concentrations differed between 0.2, 0.4, 0.6, 1, and 1.5 wt % in relation to the solid content. Determining the slurry’s pumpability and significance of dipping or coating operations demands a detailed understanding of its rheological properties. An essential metric for evaluating the flow characteristics of various phases of matter, such as gases, liquids, and semi-solids, is viscosity [60]. In this scrutiny, measurements of viscosity were carried out in conjunction with considerations of flow behavior, the effectiveness of the direct writing method, and the caliber of the sintered lines.

The study investigated the rheological behavior of mixtures with varied concentrations, as presented in Table 1, to evaluate the effect of dispersant on slurries with diverse solid contents. Utilizing a SC4-15 concentric cylinder geometry and a rotational viscometer manufactured by Brookfield LVDV-III Ultra Rheometers (AMETEK Brookfield, Middleborough, MA, USA), rheological measurements were taken at 22.4 °C. To guarantee reproducibility, slurry viscosity was measured at least three times (*n* = 3) at shear rates between 0.05 and 7 s^−1^.

#### 2.2.4. Slurry Preparation Process

Two discrete milling steps were employed in slurry preparation. The nano-sized LSCF powder was initially moistened by mixing it with a solvent (50% volume) involving terpineol and Triton dispersion. This mixture was ball-milled for 20 min in a Spex Mixer/Mill (Spex800 M- Antylia Scientific, Vernon Hills, IL, USA). In addition, a mixture of 50% solvent and 12% binder weight was heated to 100 °C for ball milling. Then, for a total of 90 min, the LSCF powder and binder fusion were combined and ball milled.

#### 2.2.5. YSZ Substrate Preparation

In this investigation, 34% of the YSZ particles in the base layer had a size range of 5–10 nm, while 66% of the particles had a particle size of 55 nm. A die with a 25 mm diameter was filled with the YSZ powder blend. The electrolyte base was established by sintering at 1350 °C for ten hours after a pressure of 10,000 lb was applied for five minutes. The sample surfaces were polished after sintering to positively affect cathode trace expansion. A sequence of sandpapers (180-, 400-, 600-, and 800-grain) were handled in this polishing method, with a minimum 15 min interval between each one under a 12-pound weight.

#### 2.2.6. Testing Procedures

The fluid’s internal resistance to flow is reflected in the viscosity of the cathode slurry. In Newtonian and non-Newtonian fluids, the correlation between shear stress and shear rate is unlike. When the temperature is fixed, the relationship for Newtonian fluids is linear and has a constant slope throughout a range of shear rates. On the other hand, non-Newtonian fluids display a fluctuating relationship in which viscosity either enhances at higher shear rates (shear-thickening) or drops at higher shear rates (shear thinning) [61]. This phenomenon was observed in the rheological properties of several slurries; the exact way in which they influenced direct writing was also recorded. Essential details about the slurry’s rheological behavior are presented below.

##### Stability of Slurry Viscosity with Respect to Time

The critical idea behind a rotational viscometer is that shear stress or torque is essential while rotating an item in a fluid. It estimates the torque expected to turn a spindle in the fluid at a set speed. During operation, this apparatus produces torque in the slurry. For slurries with differing compositions, different periods ought to achieve the constant viscosity level at a given torque. Consequently, it is imperative to establish the amount of time required to achieve a steady viscosity measurement prior to conducting rheological analyses. In this study, until a consistent viscosity was recorded, examinations were taken at 30 s intervals to ascertain the times desired for the viscosity to stabilize at a constant shear rate of 22.4 °C.

##### Effect of Shear Rate and Shear Stress Variation on Slurry Viscosity

Once stable times for various slurries were determined, viscosity was evaluated at discrete spindle speeds to examine the rheological characteristics of probable cathode slurries. With this process, the viscosity of the slurry was recorded by declining the shear rate from its greatest value until stability was reached. RheocalcT software (https://store.brookfieldengineering.com/rheocalct-software-standard-edition/) was used for the plotting and analysis of the shear rate against viscosity as well as the analysis of power law and other fluid models, in addition to Herschel–Bulkley and others.

#### 2.2.7. Direct Writing Cathode Lines on the YSZ Substrate

A 100 µm nozzle was utilized to dispense cathode slurries. For individual slurry variants, cathode traces of 10 mm length—four lines per slurry type—were generated on the YSZ substrate under the following conditions: nozzle-to-substrate gap of 100 µm; nozzle velocity of 0.5 mm/s; and extrusion pressures of 200 kPa, 100 kPa, and 30 kPa. Based on the lowest possible pressure essential to extrude a given slurry composition—200 kPa for slurries with 60% solids, 100 kPa for those with 50% solids, and 30 and 15 kPa for those with 40% solids, respectively—direct-writing pressures were preferred. Moreover, 50% solid content slurries were extruded at 200 kPa to compare the direct-writing efficacy of the two mixtures. Similar to this, 40% solid slurries were extruded at 100 kPa to compare the contents with 50% and 40% solids. However, 40% solid slurries were not dispensed at 200 kPa while the resulting line width was considered very high for the manufacturing of cathode electrodes.

##### Evaluating Width and Height of the Line

Using a Zeiss microscope, cathode trace observations were fabricated. The cathode lines were photographed from both the top and side profiles. Image-Pro Plus software version 11 was employed to determine the height and width of these lines both before and after they were sintered. As the average height of the profile peaked, the line height was documented.

## 3. Results

### 3.1. Stability of Slurries with Respect to Time

**Figure 2:**
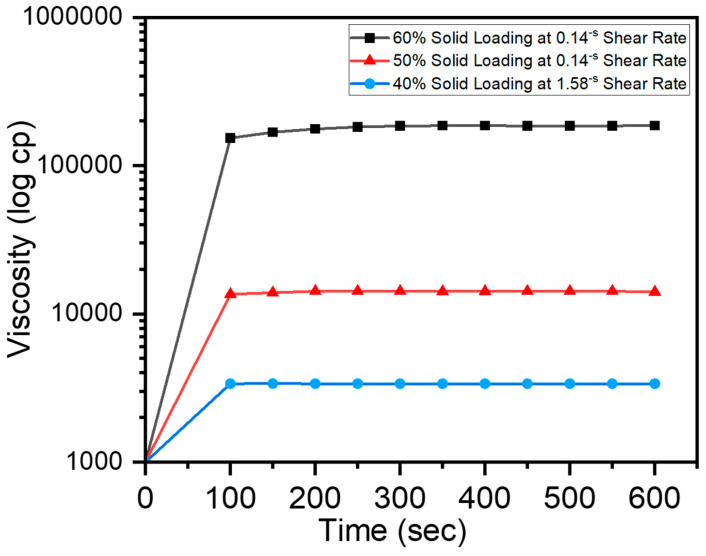
Viscosity plots of different solid loading slurries with a 0.2% dispersant and a 12% binder.

Due to its high viscosity, the slurry with a 60% solid load stabilized in 300 s. On the other hand, due to their diminished viscosities, the slurries encompassing 50% and 40% of the solid load attained stability after 200 s. The times required to reach stable viscosity rates for slurries holding 60%, 50%, and 40% solids, respectively, are shown in Figure 2. As all these measurements were obtained at the constant temperature of 22.4 °C, it is obvious that the viscosity of each slurry remained constant throughout the experiment.

### 3.2. Rheological Characteristics of Slurries

The rheological behavior of slurries was assessed by observing the apparent viscosity obtained from the shear rate versus shear stress curves at 22.4 °C. Based on various shear rates for different slurries, the shear rate varied from 0.01 s^−1^ to 7 s^−1^. The rheological data were evaluated using the Herschel–Bulkley model, a generalized model for non-Newtonian fluids. The mathematical representation for this three-parameter model is presented in Equation (1), where τ = shear stress; *D* = shear rate; *k* = consistency index; *n* = flow index; and $\tau^{o}$ = yield stress.

(1) $$\tau =\tau^{o}+kD^{n}$$

- When $\tau^{o}$ = 0 and *n* = 1, the slurry is a Newtonian fluid.
- When $\tau^{o}$ = 0 and *n* < 1, the slurry is a pseudoplastic fluid.
- When $\tau^{o}$ = 0 and *n* > 1, the slurry is a dilatant fluid (shear-thickening).
- When $\tau^{o}$ ≠ 0 and *n* = 1, the slurry is a Bingham fluid.
- When $\tau^{o}$ ≠ 0 and *n* < 1, the slurry is a viscoplastic fluid.

**Figure 3:**
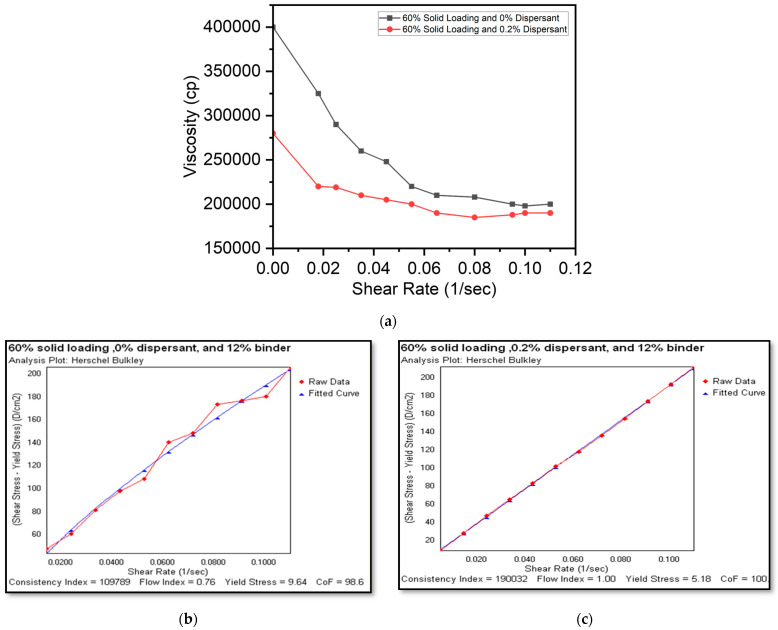
(**a**) Viscosity and shear rate plot for 60% solid loadings and a shear rate and shear stress plot (Hershel–Bulkley analysis plot) for slurries with a 60% solid loading and a 12% binder with (**b**) a 0% dispersant (viscoplastic) and (**c**) a 0.2% dispersant (Bingham).

**Figure 4:**
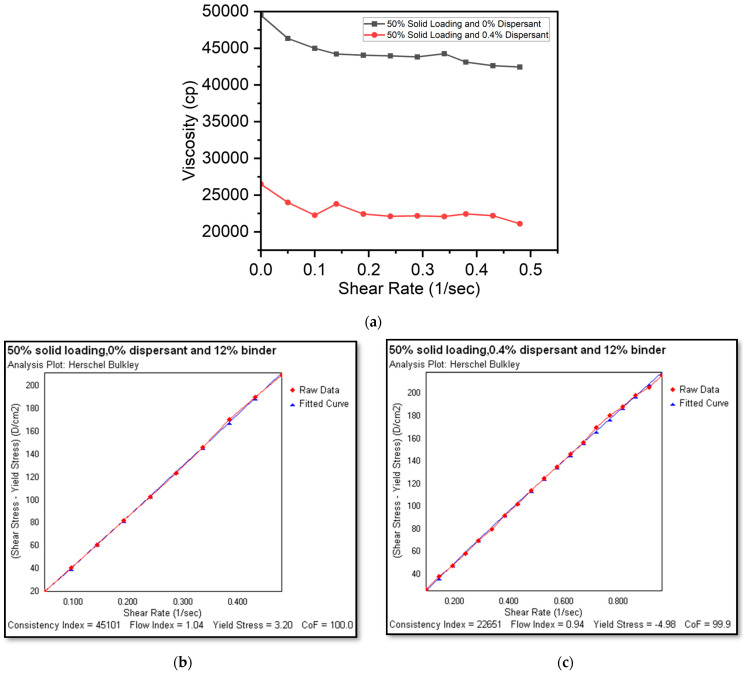
(**a**) Viscosity and shear rate plot for 50% solid loadings and a shear rate and shear stress plot (Hershel–Bulkley analysis plot) for slurries with a 50% solid loading and a 12% binder with (**b**) a 0% dispersant (Bingham) and (**c**) a 0.4% dispersant (pseudoplastic).

**Figure 5:**
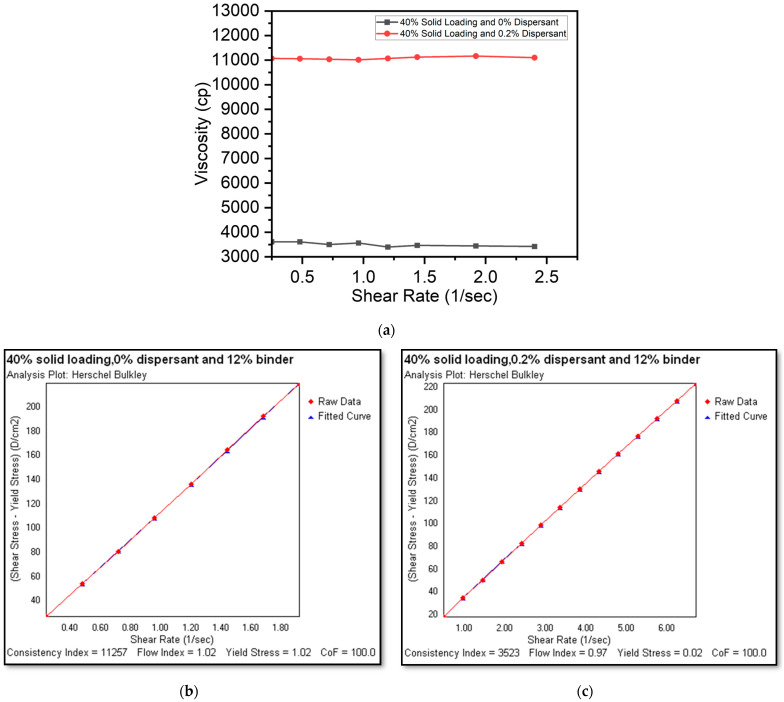
(**a**) Viscosity and shear rate plot for 40% solid loadings and a shear rate and shear stress plot (Hershel–Bulkley analysis plot) for slurries with a 40% solid loading and a 12% binder with (**b**) a 0% dispersant (Newtonian) and (**c**) a 0.2% dispersant (Newtonian).

In a static state, a Bingham or a viscoplastic fluid behaves like a solid. It takes a specified amount of force—referred to as yield stress—to initiate a flow. The flow starts as soon as this yield threshold is surpassed. Plastic fluids may then demonstrate Newtonian, pseudoplastic, or dilatant flow characteristics. Figure 3, Figure 4 and Figure 5 illustrate the viscosity–shear rate relationship as well as shear rate and shear stress plots (made possible by Hershel–Bulkley analysis) for slurries with different concentrations which highlight the inimitable flow characteristics of each slurry.

Figure 3a–c show the rheological characteristics of 60% solid loading with 0% and 0.2% dispersants, respectively. The slurry with a 0% dispersant displayed significantly higher viscosity (400,000 cP) and initial yield stress (9.64 D/cm^2^) as seen from the Hershel–Bulkley plots. However, an increase in the shear rate resulted in a lowering of viscosity indicating shear thinning (viscoplastic) fluid behavior, whereas the slurry with a 0.2% dispersant had lower viscosity (260,000 cP), initial yield stress (5.14 D/cm^2^) and marginal reduction in viscosity with increasing shear rate, indicating a Bingham fluid behavior. This can inferred from the lower flow index of 0.76 for a 0% dispersant (Figure 3b) versus a 1.0 flow index for 0.2% dispersant (Figure 3c) slurries, respectively. The characterization of high solid loading slurries has implications in selecting high extrusion pressures and relatively wider nozzle sizes. In contrast, higher solid loading can benefit from denser LSCF cathode traces.

Figure 4a–c show the rheological characteristics of a 50% solid loading with 0% and 0.4% dispersants, respectively. The slurry with a 0% dispersant (Figure 4b) displayed relatively higher viscosity (50,000cP) and initial yield stress (3.20 D/cm^2^) as seen from the Hershel–Bulkley plots. However, an increase in the shear rate did not result in changes to the flow index (1.04), indicating a Bingham fluid behavior, whereas the slurry with a 0.4% dispersant (Figure 4c) had lower viscosity (26,500cP) and varying viscosity with increasing shear rate, indicating a pseudoplastic fluid behavior. The 50% slurries provide an option for usage of both finer and relatively larger extrusion nozzle sizes, thus providing process parameter choices for 3D printing practitioners while depositing LSCF cathode traces.

Figure 5a–c show the rheological characteristics of a 40% solid loading with 0% (Figure 5b) and 0.2% dispersants, respectively. Both the slurries displayed Newtonian fluid behavior. However, the addition of a 0.2% dispersant (Figure 5c) had a significant influence on the reduction in slurry viscosity from 11,000 cP to 3600 cP. Lower viscosities can permit deposition at lower extrusion pressures. Moreover, finer nozzle dimensions can be employed to deposit thinner cathode electrodes. However, extrusion pressure and line deposition speeds need to be modified to ensure consistent trace width and height.

The usage of different slurry compositions mentioned above provides rheological guidance for selecting process parameters for depositing LSCF cathode electrodes. These include extrusion pressure, line speed, and nozzle dimensions, which can significantly impact the trace dimensions and subsequent SOFC operation.

### 3.3. Evaluating the Optimal Dispersant Concentration

**Figure 6:**
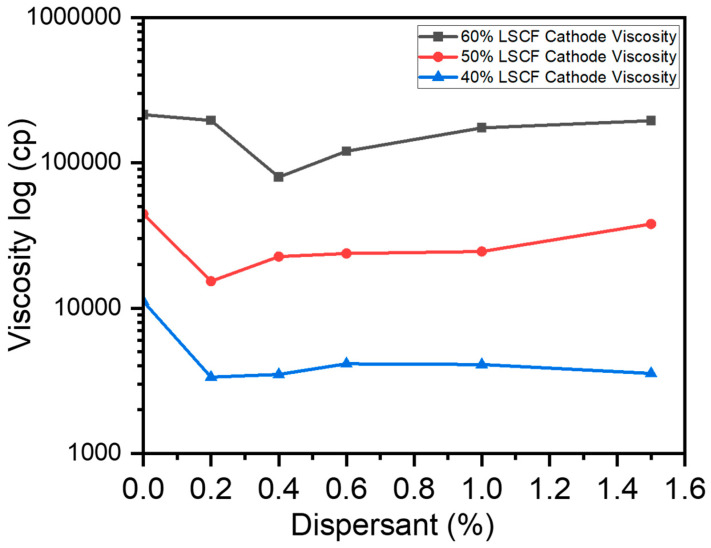
Viscosities of different LSCF solid loading slurries vs. dispersant concentration.

To evaluate how dispersants influenced the flow properties of slurries, rheology measurements were employed. Slurries that were not treated with dispersants had their viscosity evaluated in order to create a baseline. Shear stress was roughly 158 D/cm^2^, and apparent viscosities were measured when torque equaled 70%. The viscosity of the candidate slurries is plotted against the amount of dispersant in wt% of the LSCF powder employed. With a 0.4 wt% dispersant concentration, the Triton dispersant attained the lowest viscosity for slurries with a 60% solid content, decreasing it by approximately 70% when compared to the dispersion without any dispersant (Figure 6).

The optimum dispersion level for slurries including 60% solids is 0.4 weight percent. Due to its enormous adsorption on the slurry powder surfaces, the dispersant substantially reduced the Hamaker constant and improved the characteristics of the surfaces, which is why it was so effective. A dispersant concentration ranging from 0.2 to 0.4 weight percent was acquired to produce the least viscosity for slurries with 50% and 40% solid contents (Figure 6). But when the dispersant increased over this threshold, the extra dispersant molecules that stayed liberated in the mixture caused viscosity to increase. This occurred due to interactions between the excess dispersant molecules and the solvent [58].

### 3.4. Evaluating Width and Height of Lines

The three cathode lines were deposited with the slurries on the YSZ substrate. For the 60% solid content slurry, 200 kPa was the bare minimum extrusion pressure. It was feasible to extrude slurries with 40% and 50% solid contents at 100 kPa and slurries with a 40% solid content at as low as 15 kPa. Nevertheless, operating the extrusion at these low pressures caused obstacles during the direct-writing process. As a result, the measurements recorded in the table relate to slurries with a 40% solid content at 100 kPa, a 50% solid content at 200 kPa, and a 60% solid content at 200 kPa.

For 60% solid loading slurries, under 200 kPa, the range of line width was 190 µm −330 µm. For 50% solid loading slurries, under 200 kPa, the range of line width was 360 µm–670 µm. For 40% solid loading slurries, under 100 kPa, the range of line width was 550 µm–1250 µm. The line width increased with the increase in the solid loading significantly. Table 2 shows the summary of viscosity and dimension without slurry composition as slurries with different solid loadings, binder concentrations, and dispersants can provide varying viscosities. This outline can be used to choose the direct writing pressure in general, based on the viscosity of the slurry.

### 3.5. Binder Removal Process

**Figure 7:**
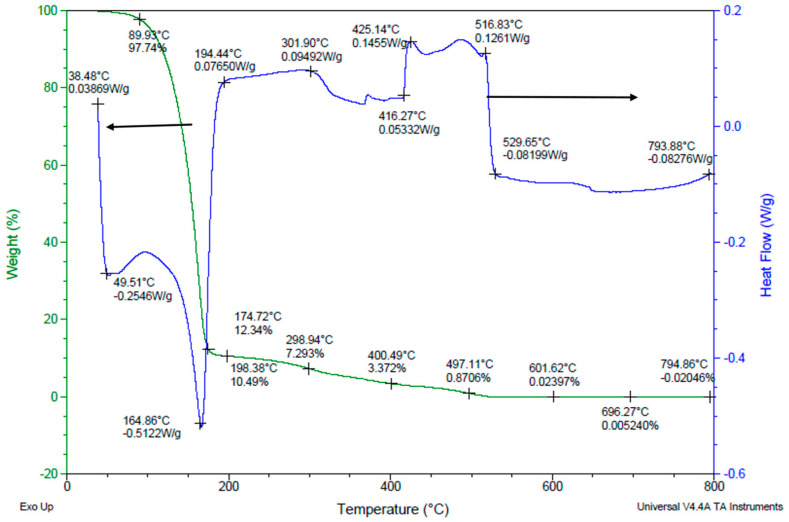
Binder decomposition process.

The sintering temperature curve was determined by the binder removal research study. Figure 7 illustrates the Thermo Gravimetric Analysis (TGA) and Differential Scanning Calorimetry (DSC) data for PVB drying at a 5 °C/min heating rate from room temperature to 800 °C. The TGA shows the changes in the weight of a specimen while its temperature is increased. The black curves represent the heating cycle from room temperature to 800 °C and a corresponding loss in binder mass at each temperature data point. DSC is a thermodynamical tool for direct assessment of the heat energy uptake, which occurs in a sample within a regulated increase or decrease in temperature. The markings in DSC graph (blue line) show both exothermic (heat-releasing) and endothermic (heat-absorbing) reactions occurring within the slurry mixture at different temperatures showing phase change in the materials and evaporation of the dispersant. An endothermic process was liable for the notable 87% mass loss that was seen at temperatures between 150 °C and 200 °C. Both endothermic and exothermic reactions were associated with the binder removal process between 200 °C and 600 °C, which led to the residue-free breakdown of the final 10% of the mass. The mass did not vary between 600 °C and 800 °C, indicating that the binder was wholly eliminated. These outcomes concur with Kim’s group research study [22].

**Figure 8:**
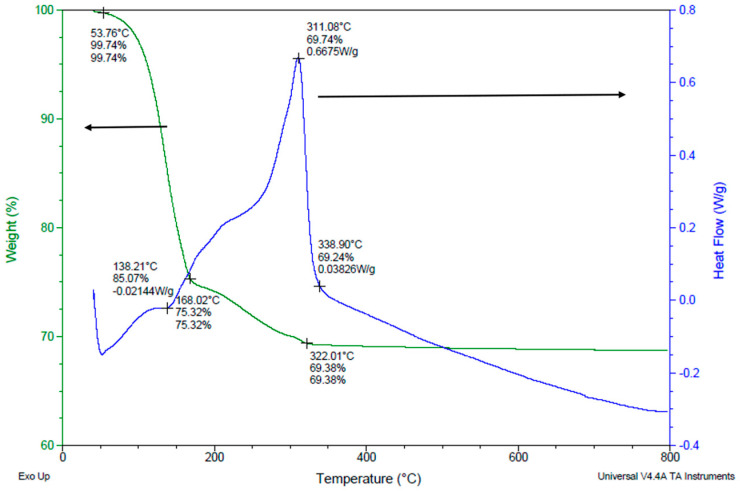
Drying process of a 60 wt% LSCF slurry with a 12% PVB, a 1.5% Triton.

The experimental results of the drying process using TGA and DSC investigations are illustrated in Figure 8. The LSCF slurry, which has a 60% solid content, a 12% binder, and a 1.5% dispersant, was dried at temperatures as high as 800 °C. Between 50 °C and 150 °C, a significant amount of the overall mass, roughly 83%, vanished. Exothermic reactions caused a continuous mass loss of approximately 20% between temperatures of 150 °C and 350 °C. The mass remained stable above 350 °C and an endothermic process occurred. There are noticeable differences in the way the binder is eliminated when blended with LSCF powder when comparing Figure 7 and Figure 8. To be more specific, Figure 8 illustrates that the slurry released a significant quantity of heat in the 150–300 °C region. With a boiling point of about 219 °C at 100 kPa, the solvent (α-terpineol) may have vaporized, which could be the cause of this event.

**Figure 9:**
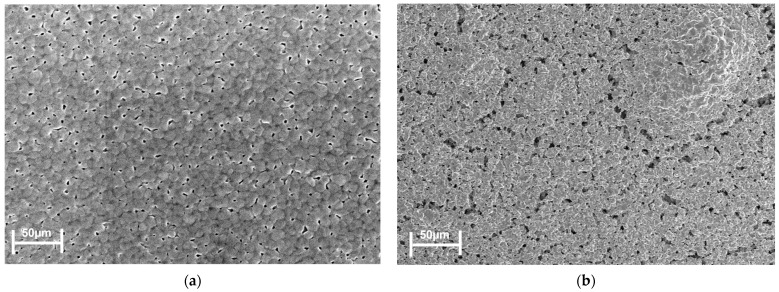
Scanning electron microscopy (SEM) images of the cathode electrode. (**a**) No cracks. (**b**) With cracks.

There were substantial cracks on the surface of the cathode electrode, as illustrated in Figure 9b, which were triggered by the fast-drying rates based on the slurry drying temperature profile that was previously described. The subsequent experiment attempted to address this by slowing down the heating rate to 3 °C per minute while holding the sintering temperature at a constant 150 °C for an hour. The revised drying regimen had modified mass loss and heat flow profile, and at 150 °C, an exothermic reaction with extra mass loss was perceived. The mass loss was broken down as follows: The mass stayed constant from 350 °C to 800 °C, losing only 17% between 150 °C and 350 °C, roughly 8% during the dwell period, and 73% between 50 and 150 °C.

**Figure 10:**
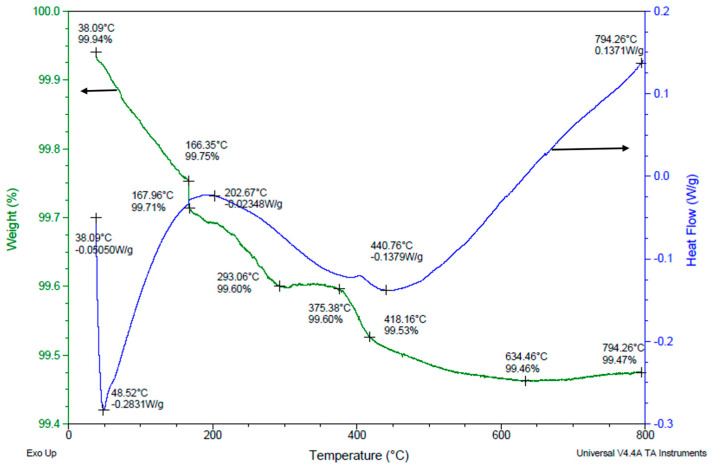
The drying process of the lines which were written by a 60 wt% LSCF, a 12% PVB, and a 1.5% dispersant slurry with dwell and ramp temperature cycling.

Figure 10 illustrates the drying process trajectory of the lines which were deposited by a 60 wt% LSCF, a 12% PVB, and a 1.5% dispersant slurry on the substrate. The mass loss was found to be 46.3% in the temperature range of 50 °C–150 °C; throughout the dwell period, it was 7.4% lower; and from 150 °C to 300 °C, it was 20.4% lower. After a 26% drop from 375 °C to 600 °C, the mass stabilized between 600 °C and 800 °C. The mass remained persistent between 300 °C and 375 °C. There was an exothermic reaction at 150 °C that led to more mass loss. Between 350 °C and 400 °C, there was a more substantial mass decrease. In this case, the fabrication of lengthy surface fractures was avoided by reducing the drying speed.

By varying the dwell, heat ramp-up, and cooling ramp-down cycles for curing the electrode lines a suitable drying process of the LSCF line was accomplished. Based on the dispersant evaporation and binder decomposition findings, an optimal sintering profile was determined as follows. The heating cycle began at room temperature to 150 °C at a rate of 3 °C/min. This was followed by a dwell period of 1 h. The heating was further initiated to 800 °C at a rate of 3 °C/min followed by a subsequent dwell period of 1 h. The final heating cycle was initiated through 1300 °C at a rate of 5 °C/min. The cathode trace was held at dwell for 3 h at the highest temperature. Finally, a gradual cooling cycle was performed to 50 °C at a rate of 5 °C/min. Thus, a stepwise heating and a dwell profile served as an optimal profile for crack-free and porous cathode electrodes for the SOFC.

## 4. Conclusions

In this research, a parallel-connected inter-digitized design of a micro-single-chamber SOFC (µ-SC-SOFCs) was fabricated with a direct-writing process. The cathode electrode slurry was investigated to understand and optimize the direct-write process [62]. The effects of dispersant and binder concentration on the cathode line dimensions were evaluated. Analysis of various amounts of the dispersion (Triton) was essential to observe its influence on the LSCF slurry. Higher viscosity slurries with 60% solid loading resembled the traits of Bingham and Viscoelastic fluids, which had a primitive yield stress. While the viscosity of the 50% solid loading slurries was slightly lower than that of the 60% solid loading ones, they demonstrated properties comparable to those of pseudoplastic and shear-thickening fluids. Of all the slurries explored, those with 40% solid loading had the minimum viscosity and mostly posed Newtonian and pseudoplastic characteristics. Triton was found to be an exceptional dispersant for cathode slurries; the optimal concentration for advancing the dispersion in various slurries was roughly 0.2–0.4% of the LSCF solid content. Further, the binder removal process was investigated because it has a significant impact on fuel cell microstructure. The sintering temperature profile was determined from the binder removal process in this study. Based on the binder decomposition results, the sintering profile was determined as follows: Temperature: room to 150 °C; rate: 3 °C/min; dwelling: 1 h; heating: to 800 °C; rate: 3 °C/min; dwelling: 1 h; heating: to 1300 °C; rate: 5 °C/min; dwelling: 3 h; and cooling: to 50 °C; rate: 5 °C/min. This research lays the foundation for developing optimal process parameters and sintering cycles for the deposition of high-fidelity cathode electrodes for micro single-chamber solid oxide fuel cells for green energy.

## Figures and Tables

**Table 1:** Slurry concentration combinations.

Solid Loading (wt%/v)	Dispersant (% of Solid Loading wt)	Binder (% of Solid Loading wt)
40%	0.0	12%
40%	0.2	12%
40%	0.4	12%
40%	0.6	12%
40%	1.0	12%
40%	1.5	12%
50%	0.0	12%
50%	0.2	12%
50%	0.4	12%
50%	0.6	12%
50%	1.0	12%
50%	1.5	12%
60%	0.0	12%
60%	0.2	12%
60%	0.4	12%
60%	0.6	12%
60%	1.0	12%
60%	1.5	12%

**Table 2:** Summary of viscosity and line dimensions.

Pressure (kPa)	Viscosity (kcp)	Line Width before Sintering (µm)	Line Height before Sintering (µm)
200	150–220	180–260	20–36
200	80–150	280–330	30–40
200	20–45	360–500	23–40
200	10–20	>600	Around 100
100	10–15	Around 600	Around 190
100	3.2–5	>800	40–100

## Data Availability

The original contributions presented in the study are included in the article, further inquiries can be directed to the corresponding author.
